# Alterations of estrous cycle, 3β hydroxysteroid dehydrogenase activity and progesterone synthesis in female rats after exposure to hypobaric hypoxia

**DOI:** 10.1038/s41598-020-60201-4

**Published:** 2020-02-26

**Authors:** Snigdha Shaw, Utkarsha Kumar, Gopinath Bhaumik, M. Prasanna Kumar Reddy, Bhuvnesh Kumar, Dishari Ghosh

**Affiliations:** High Altitude Physiology Lab, Defence Institute of Physiology and Allied Sciences, Delhi, India

**Keywords:** Reproductive biology, Risk factors

## Abstract

The underlying mechanism regulating hypoxia induced alteration in female steroid hormones is first time explored in this study. To understand the mechanistic approach, female Sprague- Dawley rats were exposed to acute and chronic hypobaric hypoxia (282 mm-Hg, ~7620 m, 6 hours, 3 and 7 days). Estrous cycle, body weight, plasma progesterone and estradiol levels, morphology, histology and two key steroidogenic enzymes: 3ß hydroxysteroid dehydrogenase (HSD) and 17ß HSD activity of ovary and adrenal gland were studied. A persistent diestrous phase and a significant decrease in body weight were found in chronic hypoxia groups. Histological study suggested degenerative changes in ovarian corpus luteum of 7 days chronic hypobaric hypoxia (7CHH) group and a declined percentage of adrenocortical cells in 3 days chronic hypobaric hypoxia (3CHH) and 7CHH groups. Plasma estradiol level was unaltered, but progesterone level was decreased significantly in all hypoxic groups. Ovarian 3ß HSD activity was decreased significantly with increasing days of hypoxic treatment along with a significantly low adrenal 3ß HSD activity in 7CHH. In conclusion, hypobaric hypoxia causes a state of low circulatory progesterone level in females likely due to the degenerative changes in the female ovarian and adrenal tissues together with low steroidogenic 3ß HSD enzyme activity.

## Introduction

According to the existing literatures hypobaric hypoxia seems to be a challenge for people visiting high altitude (HA), as the reproductive system of visitors from low altitude are more affected than the natives^1–6^. Although, the effects were seemed to be temporary and recovered with time after returning to low land^3,4,7–9^. A decrease in testosterone level^2–4,7,8,10^ along with disturbed spermatogenesis, sperm count and motility^3–5,7,11–13^ in men as well as animals at HA was reported. In case of females, previous available studies were mostly concerned with fertility at HA, in which the reason of low fertility rate had been described as hypoxia along with sociological factors^14–17^. As per reports, the reproductive hormonal profile at HA were also varied from sea level^18^. A higher estradiol (E2), decreased progesterone (P) level in Peruvian HA natives (4340 m) and a decreased prolactin and P levels in sea level women, trekked 14 days at 2800 m–5050 m were found^6,19^. The serum FSH (Follicle Stimulating Hormone) level was higher in late luteal and early follicular phase in women living at 4340 m than at sea level (150 m)^20^. On the contrary, no change in salivary P, E2 and length of menstrual cycle of Andean population (4000 m) were found, compared to other available studies on HA dwellers^21,22^. The length of menstrual cycle was found to be longer in HA residents^19,23^ whereas disturbed cycle and shorter cycle length were reported in female lowlanders after returning to sea level from their stay at 14,110 ft (4300 m) for 2 months^1^. A deleterious effect of hypobaric hypoxia on reproductive system of female rats was also reported. Regular estrous cycle is an indicator of successive ovulation; cyclicity of which was found to be disrupted in rats during 1–10 days exposure to 4267 m and recovered after returning to sea level^24^. An inhibited ovulation in female rats exposed to 5182 m^25^ and diminished reproductive outcome and abnormality in reproductive parameters of both female and male rats in another study was found after chronic exposure to intermittent hypoxia^12^. Hence, these studies suggest that hypoxia affects reproductive hormonal profile, but the mechanism responsible for these changes is still unexplored. The two steroid hormones: E2 and P are accountable for growth, development and functioning of ovarian follicles and maintenance of female reproductive cycle. Steroidogenic 3ß hydroxysteroid dehydrogenase (HSD) enzyme mediates oxidative conversion of pregnenolone to progesterone^26^ and is located in rat ovary and adrenal tissue^26,27^. The 17ß HSD is basically belongs to ovarian granulosa cells^28^ and is responsible for synthesis of E2^29^. The response of these two enzyme activity to other stressors were reported^30,31^ in which the consequence was alteration in respective hormonal levels in the circulation.

Since, hypobaric hypoxia alters female reproductive hormonal profile, the probable mechanism responsible for these changes need to be assessed. Therefore, the present study investigated the effect of exposure to hypobaric hypoxia for shorter and longer duration on steroidogenic enzyme activity, ovarian and adrenal morphology and the resulting synthesized steroid hormones in female rats.

## Materials and Methods

### Ethical approval

Animal Ethical Committee of the Institute in accordance with Committee for the Purpose of Control and Supervision of Experiments on Animals (CPCSEA), of the Government of India has approved the study (IAEC No. DIPAS/ IAEC/ 2017/ 09). The ARRIVE (Animal Research: Reporting of *In Vivo* Experiments) guidelines for reporting animal research was followed^32^.

### Animals

Non-mated female Sprague-Dawley rats (age: 10–12 weeks, body weight: 200–230 gm) were housed at normoxic condition and hygienically maintained under 12 h light-12h dark condition (temperature 24 ± 2 °C, humidity 55% ± 2%) in the Institute’s animal house. Food and water were given *ad libitum*.

### Treatment groups

The rats of experimental groups were exposed to normoxia and hypoxia following an established protocol^33^ and devided into four groups. (a) Control group (C): rats were kept in Institute’s animal house and were not exposed to hypobaric hypoxia (normoxic control) (25 ± 1 °C, 55% ± 2%, 12 h diurnal cycle); (b) Acute hypobaric hypoxia (AHH) group: rats were exposed to a simulated altitude of 7620 m (282 mm-Hg) for 6 hours; (c) 3 days chronic hypobaric hypoxia (3CHH) group: rats were first acclimatized to a simulated altitude of 4570 m (428 mm-Hg) for 24 hours and then were exposed to 7620 m (282 mm-Hg) for 3 days; (d) 7 days chronic hypobaric hypoxia (7CHH) group: rats were first acclimatized as before and then were exposed to 7620 m (282 mm-Hg) for 7 days. A specially designed animal decompression chamber with controlled partial pressure of oxygen was used for hypobaric hypoxia exposure. The temperature, humidity, light-dark cycle and air flow inside the chamber was maintained as 25 ± 1 °C, 55% ± 2%, 12 h light- 12 h dark and 0.5 L/ min/ animal respectively, similar to the conditions of rats kept in normoxia control group. The chamber was opened for 15–20 minutes each day to change the bedding, replenish the diet, measure the body weight of the rats and to take the vaginal smear for identification of estrous cycle phases.

### Estrous cycle recording

The vaginal smear of each rat was taken with 0.9% sodium chloride (NaCl) solution, placed on a glass slide and allowed to dry. The dried smear was then fixed with 70% ethanol and stained with 0.5% methylene blue (M6900 Sigma Aldrich) solution, following an established protocol^34^ and was observed under a light microscope at a magnification of 40X to identify the phases: proestrous, estrous, metestrous and diestrous phase^34^. The rats with proper progressive cycle (a total duration of 4–5 days) were considered having regular estrous cycle and further used for hypoxia exposure.

### Blood and tissue collection

The control group as well as hypoxic group rats were weighed and anesthetized with ketamine/ xylazine (70 and 6 mg/kg, i.p., respectively) immediately after exposure. Blood was collected and the rats were perfused with ice-cold phosphate buffered saline (PBS). The ovary and adrenal glands were excised, trimmed of adherent fat, washed in PBS and weighed. One ovary and adrenal gland were fixed in buffered formaline solution for histological study and another one was snap-freezed and kept at −80 °C for enzyme activity study.

### Plasma separation

The collected blood was centrifuged (DLAB, DM0412, United States) at 1000 × g for 15 minutes. Plasma was separated and stored at −80 °C for hormonal assay.

### Gonado-somatic and Adreno-somatic index

The weight of the ovary and adrenal were used to calculate gonado-somatic index (GSI) and adreno-somatic index (ASI) by using the following formulae^35,36^:

$$\begin{array}{rcl}{\rm{GSI}} & = & ({\rm{average}}\,{\rm{weight}}\,{\rm{of}}\,{\rm{both}}\,{\rm{of}}\,{\rm{the}}\,{\rm{ovaries}}\,{\rm{in}}\,{\rm{gm}}/{\rm{body}}\,{\rm{weight}}\,{\rm{in}}\,{\rm{gm}})\,\ast \,100\\ {\rm{ASI}} & = & ({\rm{average}}\,{\rm{weight}}\,{\rm{of}}\,{\rm{both}}\,{\rm{of}}\,{\rm{the}}\,{\rm{adrenal}}\,{\rm{glands}}\,{\rm{in}}\,{\rm{gm}}/{\rm{body}}\,{\rm{weight}}\,{\rm{in}}\,{\rm{gm}})\ast 1000\end{array}$$

### Histological study

The routine histological study of formaline fixed ovary and adrenal tissues from each group (n = 3) were performed by dehydrating, clearing and by embedding the tissues in the paraffin block. 5 µm serial sections were cut transversely using rotary microtome and were placed on glass slides and stained with eosin-haematoxylin and was observed under a light microscope. Photomicrographs were taken using an inbuilt CCD Color Camera (Dewinter), in Dewinter microscope (DEW/ 182). Images were captured at 40X, 100X and 400X magnifications. Adrenal cortical cell counts were made by using ImageJ cell counter software (NIH, United States). To accomplish the counting, three layers of adrenal cortex were captured separately at 400X objective lens. The pictures were converted to grey scale by setting ImageJ type 16 bit. Then the haematoxylin stained cells were manually counted by ImageJ plugins cell counter.

### Hormonal assay

The plasma E2 and P levels were measured by using all species estradiol ELISA kit (LSBio, Inc. Catalog No.LS-F5297) and rat progesterone ELISA kit (Biovendor Research and Diagnostics Products, Catalog No. RTC008R) respectively, following the manufacturer’s protocol. According to the kits the sensitivity to detect E2 was 4.45 pg/ ml and 0.04 ng/ ml for P. Absorbance was measured at 450 nm for both the hormones, by using a multireader (Tecan Infinite 200 PRO, Switzerland).

### Protein concentration estimation

The protein concentration of ovary and adrenal tissue were measured to express the steroidogenic enzyme activity. Bradford’s method^37^ by taking BSA (A2153 Sigma) as standard, was used for the measurement of protein concentration at 595 nm (Tecan Infinite 200 PRO, Switzerland).

### Enzyme assay

Enzyme activity of 3ß HSD and 17ß HSD was measured using a pre-established method^30^. Freshly collected ovary and adrenal tissues were homogenized using a homogenizing solution (1 ml/100 mg of tissue) containing glycerol, 5 mM potassium phosphate (60350 Sigma), 1 mM EDTA (E5134 Sigma) and distilled water and centrifuged at 10,000 × g for 30 mins at 4 °C. The supernatant was collected and used for both the enzyme activity measurements. For 3ß HSD activity, 100 µM tetrasodium pyrophosphate (P8010 Aldrich), 1 ml of 0.5 mM NAD (93205 Sigma) and 30 µg 17ß-estradiol (E8875, Sigma) were added along with 100 µl of the sample. For 17ß HSD activity, 440 µM tetrasodium pyrophosphate, 1.35 µM NAD, 0.3 µM 17ß-estradiol and 5% Bovine Serum Albumin (BSA) (A2153 Sigma) were added along with 100 µl of the sample. The kinetic assay was measured by using a multireader (Tecan Infinite 200 PRO, Switzerland) for 3 minutes (15 seconds interval) at 340 nm (1U = change in absorbance of 0.001/ min) and expressed as enzyme activity/ min/ mg protein.

### Statistical analysis

Data were expressed as mean ± SD. The exposed groups were compared to control group individually by using t-test (V, 6.01; Graph Pad Prism, San Diego, CA, United States) for body weight, gonado-somatic and adreno-somatic index, plasma estradiol level, plasma progesterone level, ovary and adrenal 17ß HSD and 3ß HSD activity. All experiments were performed twice on 6 animals in each group. Level of significance was tested at *p < 0.05, **p < 0.01 and ***p < 0.001 level and ^ns^p > 0.05.

## Results

### Estrous cycle

**Figure 1:**
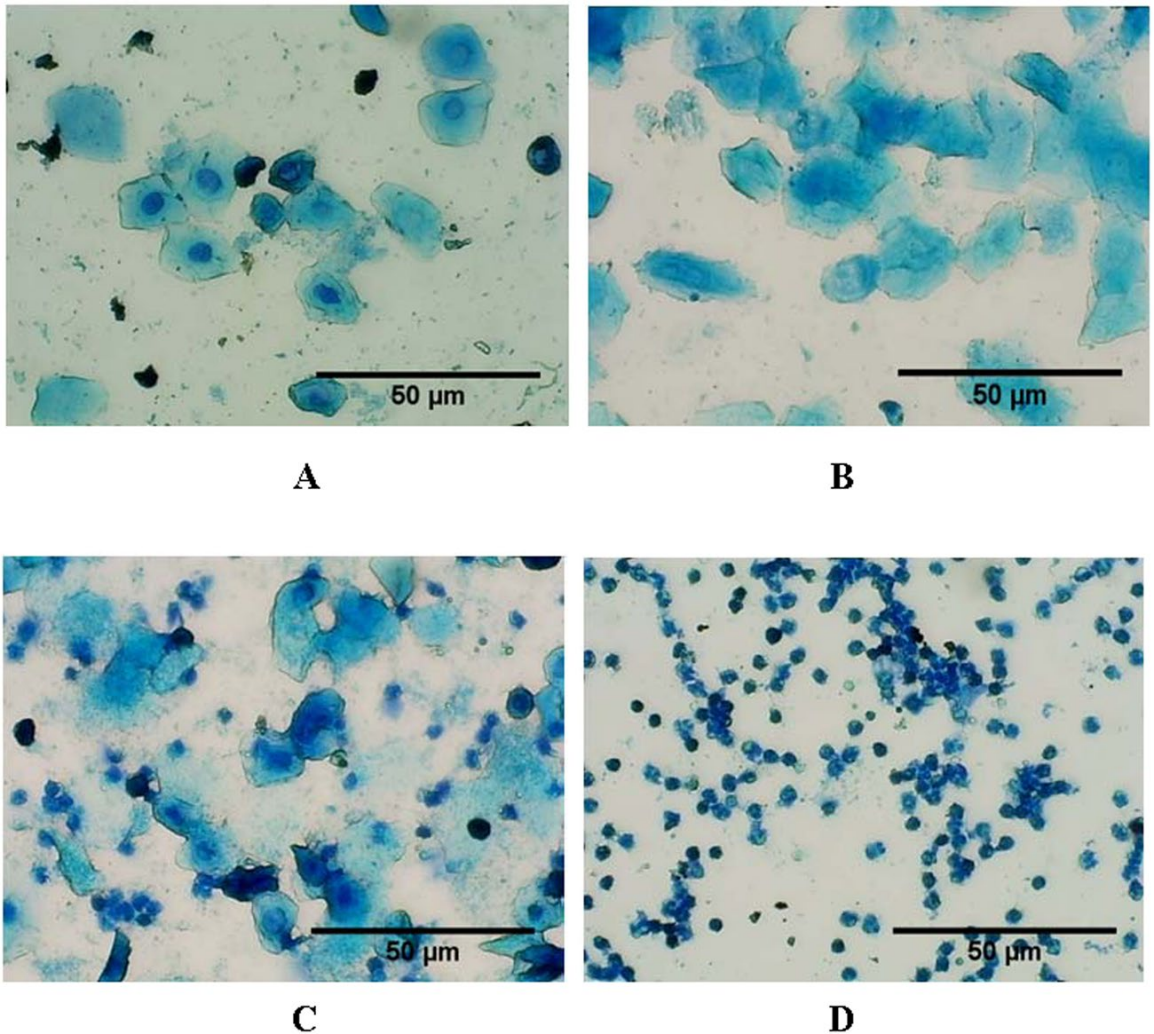
Photomicrographs of vaginal smear of rats showing four phases of estrous cycle (40X) in normoxic control rat. (**A**) Proestrous phase: nucleated epithelial cells, (**B**) Estrous phase: non- nucleated cornified cells, (**C**) Metestrous phase: nucleated epithelial cells, non- nucleated cornified cells and leukocytes, (**D**) Diestrous phase: leukocytes.

Each phase of estrous cycle was identified by presence of three types of cells^34^: numerous nucleated epithelial cells in proestrous phase; non- nucleated cornified cells in estrous phase; nucleated epithelial cells, non- nucleated cornified cells and leukocytes in metestrous phase and plenty of leukocytes in diestrous phase (Fig. 1). The vaginal cytology of control (C) group animals displayed regular estrous cycle characterized by progressive change of phases and having a total span of 4–5 days. The rats of 3CHH group showed normal cyclicity during their exposure period (Table 1a). However, the estrous cyclicity of 7CHH exposed rats exhibited a halt in diestrous phase, started from 4^th^ ± 1 day of the 7 days hypoxia exposure and on the day of sacrifice (i.e., the 7^th^ day of hypoxic exposure), most of the rats were found in the diestrous phase (Table 1b). The AHH group animals had no such changes.

**Table 1:** (a) Day-to-day recording of estrous cyclicity of rats in 3 days hypoxia exposed group and (b) Day-to-day recording of estrous cyclicity of rats in 7 days hypoxia exposed group.

Animals	Day −1^a^	Day 0^b^	Day 1^c^	Day 2	Day 3	Day 4	Day 5	Day 6	Day 7
**(a) Day-to-day recording of estrous cyclicity of rats in 3 days hypoxia exposed group**									
Rat 1	Estrous	Metestrous	Diestrous	Proestrous	Estrous				
Rat 2	Proestrous	Estrous	Metestrous	Diestrous	Diestrous				
Rat 3	Proestrous	Estrous	Metestrous	Diestrous	Proestrous				
Rat 4	Diestrous	Estrous	Metestrous	Diestrous	Proestrous				
Rat 5	Metestrous	Diestrous	Proestrous	Estrous	Metestrous				
Rat 6	Diestrous	Proestrous	Estrous	Metestrous	Diestrous				
**(b) Day-to-day recording of estrous cyclicity of rats in 7 days hypoxia exposed group**									
Rat 1	Metestrous	Diestrous	Estrous	Diestrous	***Diestrous***	***Diestrous***	***Diestrous***	***Diestrous***	***Diestrous***
Rat 2	Estrous	Metestrous	Metestrous	Diestrous	***Diestrous***	***Diestrous***	***Diestrous***	***Diestrous***	***Diestrous***
Rat 3	Proestrous	Metestrous	Diestrous	proestrous	Estrous	Metestrous	Diestrous	***Diestrous***	***Diestrous***
Rat 4	Metestrous	Diestrous	Estrous	Metestrous	Diestrous	***Diestrous***	***Diestrous***	***Diestrous***	***Diestrous***
Rat 5	Diestrous	Estrous	Metestrous	Diestrous	Proestrous	Estrous	Metestrous	Diestrous	***Diestrous***
Rat 6	Estrous	Metestrous	Diestrous	Proestrous	Metestrous	Diestrous	***Diestrous***	***Diestrous***	***Diestrous***

^a^Before the exposure, ^b^Acclimatization, ^c^Start of CHH exposure.

### Body weight and organ weight

Table 2 depicted the changes in body weight in chronic hypoxia exposed groups. CHH resulted in a substantial significant decrease (p < 0.05) in body weight on the 3^rd^ day of exposure though food and water were provided plenty, and then recovered on the 7^th^ day (but still significantly lower than the control body weight) when compared to body weight on day 0 i.e, the day before the start of the exposure. Table 3 showed the result of Gonado-somatic index (GSI) and Adreno-somatic index (ASI) among groups. No significant alterations were found in ASI, but the GSI of 3CHH group was significantly low, compared to control group (p < 0.05).

**Table 2:** Changes in body weight of experimental rats during chronic hypoxia exposure.

Parameter	Day 0	Day 3	Day 7
Body weight (gm)	209 ± 19.55	169.6 ± 24.18*	175.2 ± 23.26*

Data is presented as Mean ± SD, n = 6, *p < 0.05, ^ns^not significant.

**Table 3:** Changes in GSI and ASI in control and hypoxia exposed rats.

Parameters	Control	Acute	3 CHH	7 CHH
GSI	0.041 ± 0.006	0.040 ± 0.007^ns^	0.033 ± 0.006*	0.041 ± 0.005^ns^
ASI	0.26 ± 0.020	0.24 ± 0.025^ns^	0.26 ± 0.019^ns^	0.26 ± 0.017^ns^

Data is presented as Mean ± SD, n = 6, *p < 0.05, ^ns^not significant.

### Histopathological alterations in the ovary and adrenal gland

**Figure 2:**
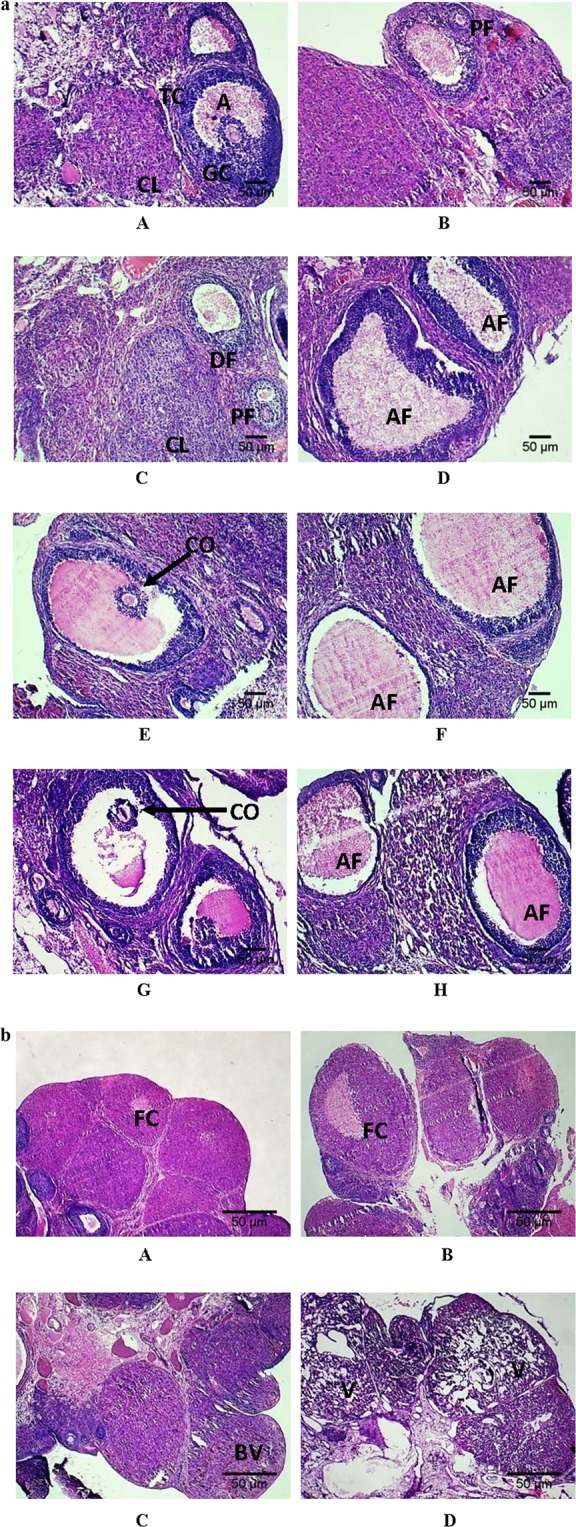
(**a**) Photomicrographs of paraffin-embedded hematoxylin- eosin stained ovary sections (100X) showing the structural changes in the follicles of normoxic control (A, B), AHH (C, D), 3CHH (E, F) and 7CHH (G, H) groups. A: antrum, TC: thecal cells, GC: granulosa cells, CL: corpus luteum, PF: primary follicle, DF: developing follicle, CO: cumulus oophorus, AF: atretic follicle. (**b**) Photomicrographs of paraffin-embedded hematoxylin- eosin stained ovary sections showing the structural changes in the corpus luteum (40X) of normoxic control (A), AHH (B), 3CHH (C) and 7CHH (D) groups. FC: follicular cavity, BV: blood vessels, V: vacuolization.

**Figure 3:**
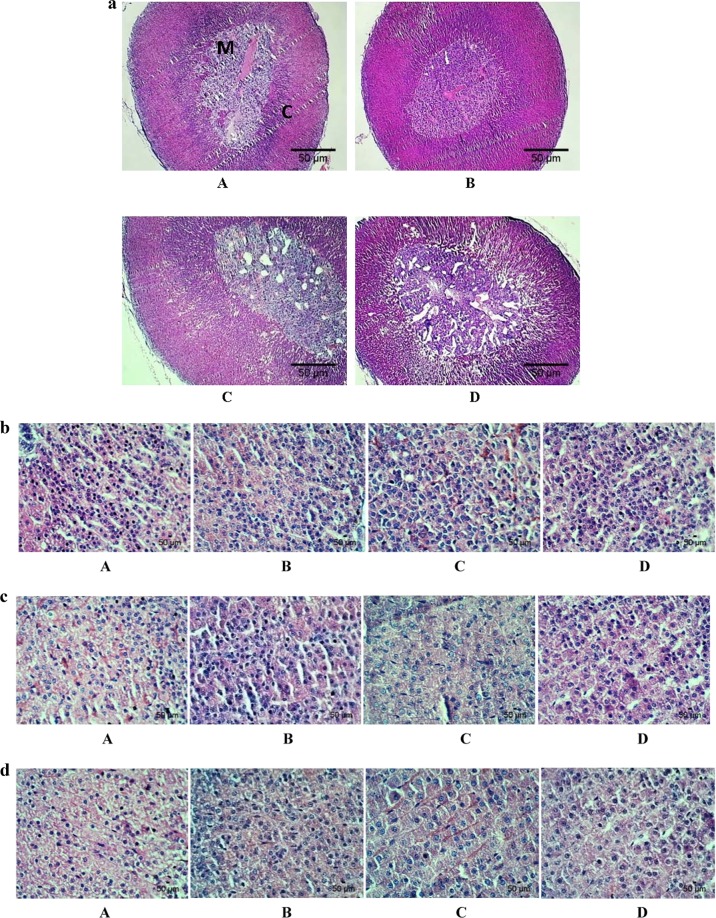
(**a**) Photomicrographs of paraffin-embedded hematoxylin- eosin stained adrenal gland sections (40X) showing the structural changes of normoxic control (A), AHH (B), 3CHH (C) and 7CHH (D) groups. M: medulla, C: cortex. (**b**) Photomicrographs of paraffin-embedded hematoxylin- eosin stained zona reticularis layer of adrenal cortex (400X) in normoxic control (A), AHH (B), 3CHH (C) and 7CHH (D) groups. (**c**) Photomicrographs of paraffin-embedded hematoxylin- eosin stained zona fasciculata layer of adrenal cortex (400X) in normoxic control (A), AHH (B), 3CHH (C) and 7CHH (D) groups. (**d**) Photomicrographs of paraffin-embedded hematoxylene- eosin stained zona glomerulosa layer of adrenal cortex (400X) in normoxic control (A), AHH (B), 3CHH (C) and 7CHH (D) groups.

The histological analysis of ovary and adrenal gland in comparison to control group were presented in Figs. 2a, b and 3a–d. The histological study of the ovary was done regarding two features of it: follicles (100X) and corpus luteum (CL) (40X). Figure 2a showed ovarian follicle (100X) having granulosa cells, thecal cell lining, ovum, antrum and cumulus oophorus. Normal antrum with intact oocyte, surrounded by granulosa cells and theca cells were present in control group (Fig. 2a A). The arrangement of granulosa cells were found to be dispersed and the thecal cell lining became less dense in 3CHH and 7CHH groups. The cumulus oophorus started to disperse in the 3CHH and this change was more evident in the 7CHH group, along with a bigger and less compact antrum (Fig. 2a,E,G). Atretic follicles were present in both the acute and chronic hypoxic groups (Fig. 2a,D,F,H), but 7CHH possessed the highest numbers.

A compact structure of CL, having a former follicular cavity in the center, was seen in control and acute hypoxic groups (Fig. 2b,A,B). A newly formed CL, containing blood vessels along with an old CL was observed in the 3 days hypoxia exposed group (Fig. 2b,C). Figure 2b,D illustrated degenerated CL in 7CHH group, having numerous vacuoles in the luteal cells.

Figure 3a revealed the structure of adrenal gland in hypoxic groups, compared to normoxic control group (40X). The control and the acute group showed intact medulla, having blood vessels and was surrounded by the adrenal cortex. Some vacuoles were found in medulla and to some extent in cortical cells of the 3CHH group. The medulla and cortex part of the 7CHH group was found to be more disintegrated.

The cell count of individual layers of the adrenal cortex (400X) showed variations (Fig. 3b–d). The cell number of the layer surrounding the central medulla i.e., zona reticularis (ZR), was found to be decreased by 23.41%, 31.85% and 37.58% in acute, 3 days and 7 days chronic hypoxia group respectively, in comparison to control (Fig. 3b). The cell number of intermediate zona fasciculata (ZF) layer ((Fig. 3c) was found to be decreased by 5.69% and 37.31% in acute and 3CHH groups respectively compared to normoxic control group. The 7CHH group showed a decline of 30.62% in cell count compared to control. The outer zona glomerulosa (ZG) showed a regressive number of cells with increasing duration of hypoxia, i.e., 19.42% in acute group, 20.63% in the 3CHH group and 33.81% decrease in the 7CHH group in comparison to control (Fig. 3d).

### Plasma steroid hormone levels - estradiol (E2) and progesterone (P)

**Figure 4:**
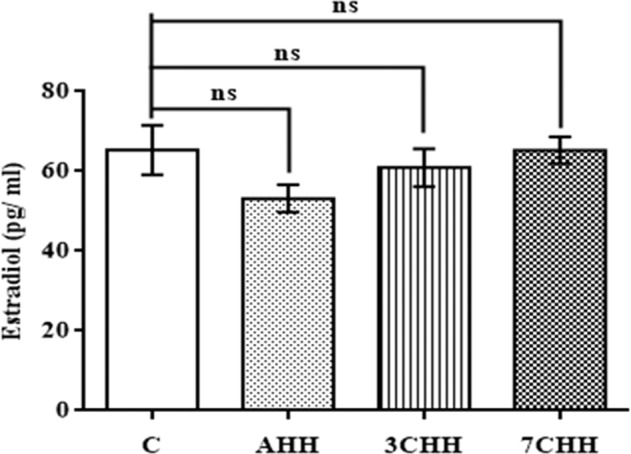
Graphical representation of alteration in plasma estradiol (E2) level in normoxic control and hypoxia exposed rats. C: normoxic control, AHH: acute hypobaric hypoxia, 3CHH: 3 days chronic hypobaric hypoxia, 7CHH: 7 days chronic hypobaric hypoxia, n = 6, ^ns^not significant.

**Figure 5:**
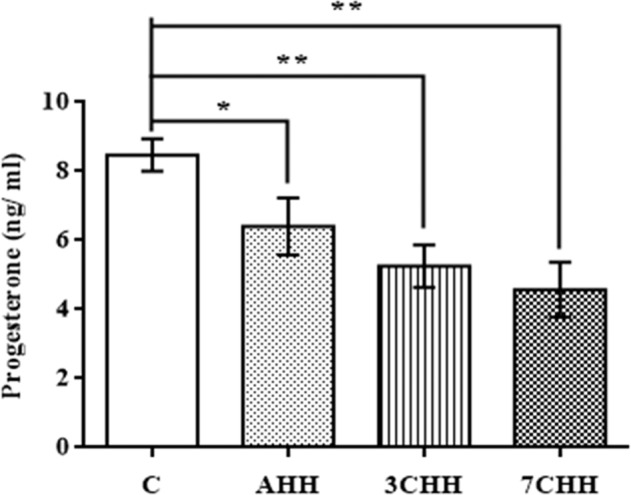
Graphical representation of alteration in plasma progesterone (P) level in normoxic control and hypoxia exposed rats. C: normoxic control, AHH: acute hypobaric hypoxia, 3CHH: 3 days chronic hypobaric hypoxia, 7CHH: 7 days chronic hypobaric hypoxia. Mean values are significantly different from control value by student’s t-test (*p < 0.05, **p < 0.01), n = 6, ^ns^not significant.

Fig. 4 have elucidated the plasma E2 level in animals after exposure to hypoxia. Both acute and chronic hypoxia exposed groups showed no significant change in E2 level in comparison to control group. The plasma P level was decreased significantly in AHH (p < 0.05), 3CHH (p < 0.01) and 7CHH (p < 0.01) groups compared to control group (Fig. 5).

### Adrenal and ovarian steroidogenic enzyme activities - 3ß HSD and 17ß HSD

**Figure 6:**
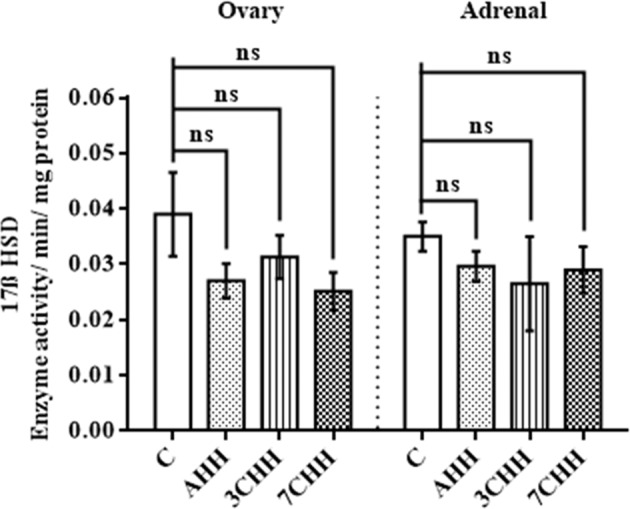
Graphical representation of alteration in ovarian and adrenal 17ß hydroxysteroid dehydrogenase enzyme activity in normoxic control and hypoxia exposed rats. C: normoxic control, AHH: acute hypobaric hypoxia, 3CHH: 3 days chronic hypobaric hypoxia, 7CHH: 7 days chronic hypobaric hypoxia, n = 6, ^ns^not significant.

**Figure 7:**
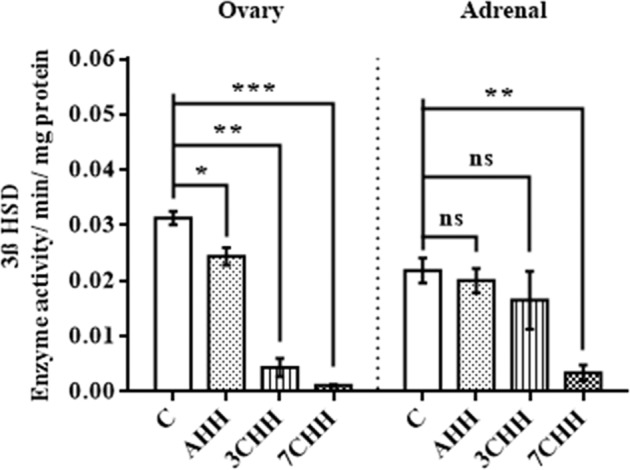
Graphical representation of alteration in ovarian and adrenal 3ß hydroxysteroid dehydrogenase enzyme activity in normoxic control and hypoxia exposed rats. C: normoxic control, AHH: acute hypobaric hypoxia, 3CHH: 3 days chronic hypobaric hypoxia, 7CHH: 7 days chronic hypobaric hypoxia. Mean values are significantly different from control value by student’s t-test (*p < 0.05, **p < 0.01, ***p < 0.001), n = 6, ^ns^not significant.

Both the ovarian and adrenal 17ß HSD activity showed no significant change in the hypoxic groups when evaluated with the control group (Fig. 6). A significant decrease in ovarian 3ß HSD activity was found in AHH (p < 0.05), 3CHH (p < 0.01) and 7CHH (p < 0.001) groups compared to control group (Fig. 7). On the other hand no significant alterations were seen in adrenal 3ß HSD activity in AHH and 3CHH groups when compared to normoxic control group. The adrenal 3ß HSD activity was found to be diminished significantly (p < 0.01) in 7CHH group in comparison to control group.

## Discussion

A handful of work was done on females at HA regarding their altered hormonal profile, but those studies were on different populations, had different durations of hypoxic exposure and had different objectives thus provided less comprehensive information^6,19,20,38–40^. Changes in circulatory hormonal level, however did not explain the underlying changes that occur in hormone-regulated female reproductive tissues. Therefore, this study was designed to illustrate the structural and mechanistic aspect of the female reproductive system, responsible for changes in sexual hormonal profile after hypobaric hypoxia exposure and we found some interesting changes which will be discussed here.

The normal reported length of rat estrous cycle is 4 days^34,41^ and we have observed similar duration of the cycle (4-5 days) in control rats of our study. In other studies, the rodent estrous cycle was reported to exhibit changes in response to atmospheric pressure, temperature and stress^24,42^. Nelson and Srebnik found an elongated diestrous or estrous phase in HA (3800 m) female native rats^43^. Donayre has also reported a diminished frequency of estrous in rats after 1–10 days exposure to HA (4267 m) that revived during 11–20 days period, prolonged estrous phase was found after 21 days and the cycle became anestrous after 90 days, which finally recovered on descent to sea level^24^. Our observation seemed to be similar with these studies, as the cycle of 7 days hypoxia exposure group became irregular due to lengthened diestrous phase.

The result of the present study showed a significant fall in body weight till 3^rd^ day of chronic hypoxia exposure and then started to regain from 4^th^ day onwards. Similar loss of rat body weight exposed to 7620 m was also reported in an another study^44^. The GSI, a scale of change in gonadal weight in relation to body weight, was found to be decreased significantly (p < 0.05) in the 3CHH group compared to control group as a consequence of low body weight. The histological study of ovarian tissue of hypoxic groups showed distinct features in comparison to the normoxic group. 3 and 7 days hypoxia exposed ovary showed regressive lining of surrounding thecal cells along with less compact granulosa cells in matured follicle. The granulosa cells surrounding the oocyte i.e., cumulus oophorus, provides nutrients to oocyte, helps it to mature and is important for fertilization^45,46^. The cumulus-oocyte complex is also important for fertilization as it prevents hardening of the zonapelucida and thus facilitates sperm penetration^47^. In this study, we found that the cumulus oophorus started to denude in the 3CHH group, which was again found to be more degenerated in the 7CHH group and therefore might interfere with oocyte maturation and ovulation process. Atresia was reported to be a result of follicular apoptosis, regulated by gonadotrophins^48^. Our observation revealed the existence of some atretic follicles in all three hypoxic groups. The CL of 7 days hypoxia exposed group depicted marked degeneration of the luteal cells. In addition, the adrenal gland of hypoxia exposed groups also portrayed interesting changes like, the medulla of 3 and 7 days hypoxia group was found to be degenerated by the formation of vacuoles and these vacuolar degenerations were more in 7 days group. The steroidogenic cells of inner ZR and ZF displayed a marked decline in percentage count than the control. The cell count of ZG or zona multiformis as termed in animals^49^, was found to be decreased in all three hypoxic groups of our study. The percentage of decrease was proportional with the increasing time of hypoxic exposure. The cells of adrenal medulla secretes epinephrine and norepinephrine, ZG of the adrenal cortex is responsible for the production of aldosterone and ZF and ZR synthesizes steroid hormones^49,50^. The ovarian CL and adrenal cortical cells are great source of progesterone and deterioration of these structures in our study might have an impact on progesterone secretion as well.

The female reproductive functions are dependent on two vital steroid hormones, viz. estrogen and progesterone^51^. In the present study, plasma steroid hormones were measured and it was found that the circulatory E2 level was unaltered, but P level was decreased significantly in all hypoxic groups compared to normoxic group. Previous literatures showed that the level of E2 remained unaltered in women exposed to stress like cold pressure test for a shorter duration^52^. But in case of animals, when female rats exposed to a longer duration (21 days, intermittently) of restraint stress, their E2 level was declined^53^, suggesting that E2 level is only affected after prolonged stressor stimuli. HA hypoxia affects female hormonal level, as Gonzales found unaltered E2 and increased follicular stimulating hormone (FSH) level in Peruvian HA female natives (4340 m) in comparison to sea level residents^20^. Another study on the same population found a declined level of leutinizing hormone (LH) and estrogen in the follicular phase of HA natives^19^. A recent study reported decreased P level in sea level natives after 14 days trekking at 5050 m, though E2, FSH and LH hormones were found to be unchanged^6^. Some studies measured the reproductive hormonal level in females at HA for phasic categorization: increased estrogen and P was found in female lowlanders in follicular phase, during their 12 day residence at HA (4300 m)^38^; a change (not significant) in luteal P level was determined on 3^rd^ and 12^th^ day of HA (4300 m) exposure, in sea level residents^39^; a declined level of luteal P and elevated E2 level in both follicular and luteal phase was reported in lowlanders during their 12 day stay at 4300 m, compared to their sea level values^40^. Apart from hypoxia other stressors also seemed to have an impact on steroid hormones. For instance, dietary cyanogens consumption, excessive iodine dosing and protozoan infection in female rodents led to both E2 and P reduction^30,31,54^. In another study toxic chemical exposure was also found to mitigate E2 in female rats^55^. After prolonged surgical stress the rat adrenal P level was found to be declined as the corticosterone level was increased^56^. In the present study, the resulting decreased P level and persistent diestrous phase could be linked together, as Haim *et al*. has reported that the P took two peaks: one in proestrous (along with E2) and another in metestrous which is of low magnitude than the former peak^57^ and an intact functional ovary is required in the late diestrous phase for entering into the proestrous phase^10^. In this study, a significant decrease (p < 0.01) in plasma P along with regressed corpus luteum and decreased adreno- cortical cell number in the 7CHH group brought about the changes in estrous cycle phases.

Following the quantification of circulatory sex hormones, the key steroidogenic enzymes, involved in synthesis of these hormones were also studied. It was found that ovarian 3ß HSD activity was decreased significantly with increasing duration of hypoxic exposure compared to normoxic condition. In addition, a significant decrease in adrenal 3ß HSD activity in 7CHH group, therefore could be associated with the diminished level of circulatory P. Liu and his team found downregulation of 3ß HSD gene, in male rats subjected to hypoxia^58^. Histochemical study of male toad testicular localization of 3ß HSD enzyme showed decreased activity along with a decrease in testicular hormone synthesis after exposure to 7,315 m in comparison to control group^59^. A previous study found a decrease in serum testosterone concentration along with a decrease in protein expression of 3ß HSD in male mice exposed intermittently to normobaric hypoxia^60^. 3ß HSD activity was also reported to be reduced significantly in female rats fed with dietary cyanogens for a longer duration^31^. Furthermore, in our study, ovarian and adrenal 17ß HSD activity in all hypoxic groups showed no significant alterations and probably resulted in unchanged plasma E2 level as well. However, the decrease in P was probable a result of regressed CL, adrenocortical cell hypoplasia and declined 3ß HSD activity, led to cessation of diestrous phase, as the amount of P was not sufficient to take a sharp peak to initiate proestrous phase in the 7CHH group. However, when some of the 7 days hypoxia exposed rats were kept at normoxic condition, it was found that the cyclic progression was resumed and the rats were no longer arrested in diestrous phase after 1-2 days exposure to normoxia (the result is not showed here).

## Conclusion

In a nutshell, it is found that chronic hypobaric hypoxia resulted in irregular estrous cycle in rats due to prolonged diestrous phase which is a consequence of mitigated plasma P level. The CL of the ovary and cells of zona reticularis and fasciculata of the adrenal cortex that are thought to produce this steroid hormone along with a decreased activity of steroidogenic enzyme 3ß HSD is responsible for low synthesis of progesterone.Therefore, the findings gathered in our study as well as the physiological regimens considered above suggested the conclusion that hypobaric hypoxia exposure for a longer duration has a detrimental effect on reproductive functions of females who have never been exposed to hypoxia before. These effects fortunately are not everlasting and may be recovered with time after returning to normoxic condition. In future, further extensive studies in this direction can be conducted to avoid the undesirable effects of sustained hypoxia on reproductive health of female lowlanders staying for longer durations at HA.
